# The interplay between home and job demands, resources, and the intention to stay in nursing: A cross-sectional study

**DOI:** 10.1016/j.ijnsa.2025.100318

**Published:** 2025-03-17

**Authors:** Jasperina Brouwer, Stéfanie André, Nienke Renting

**Affiliations:** a University of Groningen, Faculty of Behavioural and Social Sciences, Department Educational Science, Grote Rozenstraat 3, 9712 TG Groningen, Netherlands; b Radboud University, Department of Public Administration, Nijmegen School of Management, & Radboud WORKLIFE consortium, Postbus 9108, 6500 HK NIJMEGEN, Netherlands

**Keywords:** Work pressure, Work-life balance, Energy, Turnover intention, Intention to stay, Nursing

## Abstract

**Background:**

Concerns about the global nursing shortage highlight that, while increasing graduates is considered as essential, addressing retention issues is equally critical to mitigate the shortage effectively. Nurses frequently struggle to harmonize family life with their demanding profession in this predominantly female field. Finding a balance between home and job demands and resources may be crucial for staying in the profession.

**Objective:**

We aimed to obtain a better understanding of the relationships among home and job demands (parenthood, working hours, work pressure), personal resources (experience, need for autonomy, self-efficacy), contextual resources (work-life balance), and the intention to stay in the nursing profession. More specifically, we investigated how work-life balance, as a contextual resource mediated the complex interplay among home and job demands, personal resources, and the intention to stay.

**Design:**

We adopted a cross-sectional survey methodology.

**Setting(s):**

We focused on nursing professionals employed in healthcare institutions, encompassing hospitals, elderly care facilities, and home care institutions in the Netherlands.

**Participants:**

Survey respondents included 616 bedside nurses with either patient care responsibilities alone or combined with managerial responsibilities.

**Methods:**

We distributed a survey among nursing professionals in 2021. Path modeling was conducted using Mplus version 8.0.

**Results:**

The comprehensive model revealed that parenthood and the fulfilment of the need for autonomy were associated with increased energy levels, while the need for autonomy was associated with lower work pressure and higher intention to stay. Work pressure was negatively associated with work-life balance, whereas a satisfactory work-life balance was positively associated with the intention to stay. Energy was directly related to intention to stay, as well as indirectly through work-life balance. Work experience was negatively associated with the intention to stay. Controlling for gender, nurses identifying as female or non-binary experienced lower energy compared to their male counterparts.

**Conclusion:**

Work-life balance and the need for autonomy were positively associated with the intention to stay. Energy was positively associated with work-life balance and the intention to stay. Working experience was negatively associated with the intention to stay in the profession, suggesting that more experienced nurses may may see more alternatives after leaving the bedside profession.

**Tweetable abstract:**

Nurses may stay when they feel energized and experience more work-life balance. More autonomy is encouraging, whereas high work pressure discourages them. Let's support nursing needs. #Nursing

**What is already known about the topic**

• Researchers often focus on turnover intention rather than the intention to stay.
• Researchers often focus on the turnover intention of novice nurses.
• Researchers focus on either work-related factors or home factors rather than work-life balance and the link to intention to stay.

Alt-text: Unlabelled box

**What this paper adds**

• A positive work-life balance, supported by meeting nurses’ need for autonomy, was associated with their intention to stay in the profession.
• Parenthood contributed slightly to higher energy levels among Dutch nursing professionals.
• Nurses with more work experience had a lower intention to stay than nurses with less experience.

Alt-text: Unlabelled box

## 1 Background

The global shortage of nurses is escalating rapidly, posing risks to care quality and nurses’ well-being due to high workloads and pressures (WHO, 2020, 2022; Heesakkers et al., 2021). Many countries urgently need more nurses to support aging populations and increasingly complex care demands (OECD, 2021). Additionally, as nurses retire and turnover rates rise—particularly in the Netherlands, where vacancies in healthcare have outpaced other sectors—these shortages grow even more critical (CBS, 2023a; Heesakkers et al., 2021). While investments in training programs aim to increase the number of nursing graduates, retention challenges must also be addressed, as turnover rates of qualified nurses can undermine the benefits of expanded training efforts (ICN, 2022). Importantly, qualified nurses are urgently needed to train novice nurses (Cziraki et al., 2020). Insights from policy reports indicate that, without improvements in working conditions and retention strategies, expanding the workforce through training alone may not sufficiently stabilize the nursing profession (WHO, 2022).

Most researchers on nursing retention focus on turnover rather than on factors that encourage nurses to stay (De Vries et al., 2023; Lee, 2021; Xu et al., 2021). Researchers during COVID-19 identified organizational factors (e.g., social support and leadership style) and psychological factors (e.g., anxiety) as key drivers of turnover (Tolksdorf et al., 2022). Both novice and experienced nurses cited personal, working, and organizational factors as reasons for leaving (De Vries et al., 2023; Lee, 2021). While Al Yahyaei et al. (2022) concluded that working and organizational factors contributed the most to the intention to stay, Pressley and Garside (2022) found different predictors, such as job satisfaction and commitment. Thus, focusing solely on work and organizational factors overlooks the challenges that nurses face in balancing work and private life (Sirgy & Lee, 2018). Therefore, we examined home and job demands, along with personal resources, to understand their impact on work-life balance and nurses’ intention to stay.

We drew on the Job Demands-Resources model, a widely used framework for understanding retention by examining how work environments impact outcomes, such as stress and performance (Bakker & Demerouti, 2007; Tummers & Bakker, 2021). Job demands and resources have been linked to burnout and turnover intentions in nursing (De Vries et al., 2023). Moreover, the model has been extended in recent research to include home demands and resources, recognizing that family responsibilities, such as parenthood, also influence work outcomes (Ten Brummelhuis & Bakker, 2012). We applied this Work-Home Resources model, which integrates personal resources, contextual resources, and home and job demands.

In this study, we included *home and job demands*, including work pressure, influenced by working hours and parenthood. High *work pressure* often leads to exhaustion and disrupts work-life balance, contributing to turnover intentions (Riedl & Thomas, 2019). *Working hours* is an important factor to take into account, especially in combination with *parenthood*, as previous researchers have shown that gender and marital status, combined with care responsibilities and workload, may affect energy levels and work-life balance (Mirzaei et al., 2021; Nashwan et al., 2021). Earlier researchers highlighted that family obligations, when incompatible with irregular shifts or high workloads, can drive turnover intentions (Raffenaud et al., 2019). In the Netherlands, part-time work is a common strategy, particularly among female nurses, to balance professional duties with childcare or informal care (CBS, 2022, 2023b; Yerkes & Hewitt, 2019). However, the impact of part-time work on work-life balance and retention remains underexplored.

Next, we explored four *personal resources* —work experience, autonomy, self-efficacy, and energy—to assess their impact on nurses' work-life balance and intention to stay. *Work experience* may influence retention, with senior nurses, who have more work experience, tending to have a higher retention rate compared to early career nurses, who have less experience (Al Yahyaei et al., 2022; De Vries et al., 2023; Pressley & Garside, 2022). Conversely, Lee (2021) suggested that nurses with less clinical experience may also show a high intention to stay. *Autonomy*, or the freedom to make decisions, is critical for retention; unmet autonomy needs often drive nurses to leave (Kerzman et al., 2020). *Self-efficacy*—confidence in job performance—has been linked to job embeddedness which may reduce job turnover (Kim & Park, 2023). *Energy*, lastly, may play a direct role in supporting work-life balance and retention, highlighting how these resources collectively can enhance nurses' commitment to their profession.

We also included work-life balance as a *contextual resource*. Nurses generally value work-life balance and benefit from work-family policies aimed at supporting it (Moyer, 2022). When nurses face high workloads and work-family conflicts, they may consider leaving the profession as a way to protect their health and energy (Yildiz et al., 2021). A mismatch between work and private life, as shown by De Vries et al. (2023), is a common factor in turnover. Achieving a sustainable work-life balance requires a perception of fair workloads and the ability to balance professional and family responsibilities, which can help reduce stress and enhance job retention (Labrague et al., 2021).

**Fig. 1:**
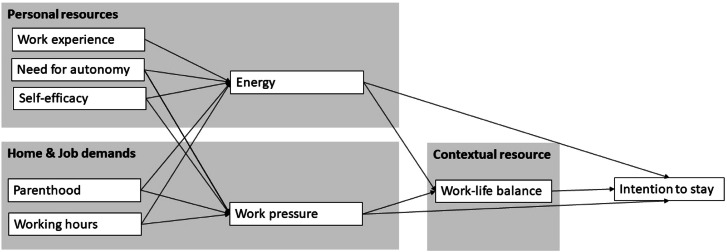
Conceptual model based on the Work-Home Resources model Note. Adapted and derived from Ten Brummelhuis and Bakker (2012).

For sustainable healthcare and a thriving nursing workforce, we need a better understanding of the interplay among home and job demands and resources, work-life balance, and the association with intention to stay in nursing. Rooted in the Work-Home Resources Model (Ten Brummelhuis & Bakker, 2012), we explored how and to what extent personal resources (work experience, need for autonomy, self-efficacy, energy) and home and job demands (parenthood, working hours, work pressure) were related to the contextual resource work-life balance and to the intention to stay, while controlling for gender. This is graphically depicted in our conceptual model in Fig. 1. By testing the conceptual model, we addressed the following two research questions: (1) To what extent and how do home and job demands, personal and contextual resources relate to work-life balance among nursing staff? (2) How do these relate to the intention to stay in the nursing profession?

## 2 Methods

### 2.1 Study design

We conducted a large-scale cross-sectional survey in the Netherlands among bedside nursing professionals, some of whom had managerial responsibilities. The survey consisted of closed questions based on validated scales.

### 2.2 Measures

Personal resources captured the following variables: work experience, the need for autonomy, self-efficacy, and energy. The full survey was not validated. Therefore, we calculated the internal consistency (Cronbach's *α*) for all the used scales after the data collection process. *Work experience* was measured with the question “How many years have you worked in the healthcare sector?'' with seven answer categories varying from "less than 6 months" to "more than 21 years”. *Need for autonomy* was a subscale with six items (Cronbach's *α* = .86), derived from Van den Broeck et al. (2010), capturing the need for having control at work; for example, with the item: "I feel free to do my job the way I think it could best be done". *Self-efficacy* was adapted to the nursing context and derived from the Motivated Strategies for Learning Questionnaire (Pintrich et al., 1991). The subscale consisted of eight items (Cronbach's *α* = .87) measuring the extent to which participants believed they could perform their job effectively. An example item is: "I feel confident in handling complex situations". Responses were recorded on a 5-point Likert scale (1 = "fully disagree” to 5 = “fully agree”). *Energy,* derived from Van Yperen and Hagedoorn (2003), measured physical energy with three items (Cronbach's *α* = .84), such as “At the end of a working day, I feel really fatigued”. The answer categories on a 5-point Likert scale ranged from 1 = “fully disagree” to 5 “fully agree”.

Home and job demands captured the variables of parenthood, working hours, and work pressure. *Parenthood* was measured with a question related to the living situation, which was recoded into two categories to distinguish between caring for children (2) or not (1). *Working hours* was measured with the answer categories: (1) = "Full-time (equal or more than 32 hours)", (2) = "Part-time (less than 32 hours)", (3) "Flexible", and (4) "Other". *Work pressure* measured how demanding the job was perceived with four items, such as "Do you have problems with the workload?", derived from Van Yperen and Hagedoorn (2003). The answer categories were scored on a 5-point Likert scale ranging from 1 = "never" to 5 = "always".

A contextual resource is work-life balance. *Work-life balance* was measured with two items (Cronbach's *α* = .88); for example, "My work and personal demands are well balanced”.

The outcome variable *intention to stay* was captured by three items (Cronbach's *α* = .92) derived from O'Driscoll and Beehr (1994). An example item is: "I intend to continue working as a nurse for at least the next 3 years”. Two items were recoded.

*Gender,* as a control variable, was asked by the question to which gender participants identified the most: (1) = "male", (2) = "female", or (3) = "non-binary".

### 2.3 Setting and data collection

The data were collected in 2021 using an online survey created with Qualtrics software (Qualtrics, Provo, UT). The survey was distributed among nursing professionals via the researchers’ personal network, nursing students’ networks, and social media platforms, such as closed professional nursing groups in the Netherlands. The inclusion criteria for participation were providing direct patient care, whether or not combined with managerial tasks, in a health care setting in the Netherlands. Participants provided written informed consent after reading the study aim (i.e., understanding nursing retention) and ethical considerations. Detailed instructions on how to complete the survey were provided in the introduction. The survey took approximately 15 minutes to complete, and participation was anonymous. Participants could complete the survey on any digital device, including smartphones. A validation request prompted participants to answer skipped questions before proceeding. However, participation was on a voluntary basis, and they could choose to skip items. Reminders about survey availability were sent via nursing networks and social media 2 and 4 weeks after the initial distribution to increase participation.

### 2.4 Sample

The sample consisted of 616 nursing professionals. All were bedside nurses (469 Registered Nurses [RNs], 103 Licensed Practical Nurses [LPNs], and 44 RNs who combined their duties with managerial responsibilities). According to the Organisation for Economic Co-operation and Development (OECD, 2021), nurses are defined as practitioners when they deliver services directly to their patients, but they can also combine this with managerial tasks. Participants were working in different settings in the Netherlands, varying from hospitals and mental health care clinics to outpatient (elderly) care.

### 2.5 Data analysis

The descriptive statistics and reliability tests (Cronbach's alpha's above), used to assess the internal consistency of the scales, were conducted in IBM SPSS 28.0. Preliminary analyses of variance (ANOVAs) were performed to examine whether mean differences existed in energy, work pressure, work-life balance, and intention to stay across RNs, LPNs, and RNs with managerial tasks.

Path analyses, performed in Mplus version 8.0 (Muthén & Muthén, 1998-2017), tested the conceptual model based on the observed variables expressed by the means of the scale items. For the indirect effects, bias-corrected bootstrapped intervals were reported (Shrout & Bolger, 2002). The model fit was assessed as good if root mean square error of approximation (RMSEA) values were below .06, standardized root mean squared residual (SRMR) at or below .08, and Tucker-Lewis index (TLI) and comparative fit index (CFI) both greater or close to .95. We considered a non-significant Chi square (*χ2*) test as a good model fit, though the *χ2* test is sensitive to the sample size (Hu & Bentler, 1999; Kline, 2023) and thus less relevant in large samples.

### 2.6 Ethical approval

The study was approved by both the ethical committee of the Faculty of Behavioral Sciences, Department of Pedagogy and Educational Sciences, University of Groningen (29-01-2020) as well as by the medical ethical committee of the University Medical Center Groningen (METc 2020/182). The study was not subject to the WMO (Medical Research Involving Human Subjects Act).

## 3 Results

### 3.1 Descriptive statistics

The sample consisted of RNs and LPNs (572; 93%) and RNs with managerial responsibilities (44; 7%). Most of them identified as female (547; 89%), followed by male (67; 11%), and other/non-binary (2; 0.3%). This was representative of the gender distribution among nurses in the Netherlands, with 84% female and 16% male (CBS, 2025). Most nurses were between 31 and 40 years old (165; 17%) and between 51 and 55 years old (117; 19%), and working part-time (394; 64%). Participants worked in various healthcare settings, mostly in hospitals (223; 36%), elderly care (121, 20%), and home care (170; 28%), whereas a minority was working in other settings, such as youth care, mental health care, care for individuals with a disability, emergency care, outpatient care, or maternity care.

Table 1 presents the descriptive statistics and their bivariate correlations. Energy, work-life balance, and need for autonomy were significantly positively related to the intention to stay, whereas work pressure was significantly negatively related. Energy and work-life balance were significantly positively related, whereas work pressure was significantly negatively related to work-life balance. The need for autonomy was significantly positively related to energy and work-life balance but negatively to work pressure. Self-efficacy was not significantly related to any of the variables.

**Table 1:** Descriptive statistics and correlations.

	Need for autonomy	Self-efficacy	Energy	Work pressure	Work-life balance	Intention to stay
Need for autonomy	1.00					
Self-efficacy	.06	1.00				
Energy	.44**	.04	1.00			
Work pressure	-.44**	.03	-.64**	1.00		
Work-life balance	.44**	.02	.49**	-.47**	1.00	
Intention to stay	.37**	-.01	.40**	-.34**	.38**	1.00
Mean	3.37	4.11	3.29	3.06	3.15	3.42
Standard Deviation	0.71	0.42	0.99	0.69	0.93	0.93
Minimum	1.00	2.50	1.00	1.40	1.00	1.00
Maximum	5.00	5.00	5.00	5.00	5.00	5.00

**p ≤ .001 *p < .05

### 3.2 Missing data

The proportion of missing data was minimal, ranging from 0.5% (for variables such as work pressure and energy) to 2.3% for the dependent variable intention to stay. Little's missing completely at random (MCAR) test yielded a non-significant result (*χ²*(6) = 6.33, *p* = .387), indicating that the data were not missing completely at random. This suggests that the missingness can be assumed as missing at random (MAR) (Missing at Random); i.e., missing data is unrelated to the dependent variable but may be related to independent variables (De Leeuw et al., 2003; Little & Rubin, 2002). Under this assumption, maximum likelihood estimation is suitable for handling missing data, as it provides unbiased parameter estimates even in the presence of missing values (Allison, 2002; Arbuckle, 1996; McKnight et al., 2007).

**Fig. 2:**
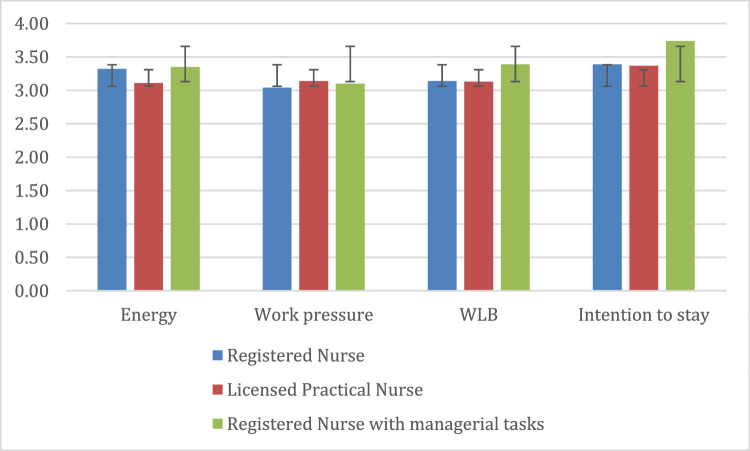
Differences in energy, work pressure, work-life balance, and intention to stay for three types of bedside nurses Note. Numbers of RNs = 469, LPNs = 103, RNs with managerial tasks = 44; WLB = Work-life balance. Error bars are based on the standard deviation.

Preliminary ANOVAs indicated no significant differences in intention to stay among bedside nurses (*M*_RN_ = 3.39; *M*_LPN_ = 3.37) compared to bedside RN with managerial tasks, *M* = 3.74; *F* (2, 599) = 2.82, *p* = .060. As illustrated in Fig. 2, RNs with managerial tasks showed higher mean levels of energy and work-life balance compared to RNs and LPNs without these responsibilities. However, these differences were not statistically significant, with *F*(2, 610) = 1,99, *p* = .137 for *energy* and *F*(2, 613) = 1,49, *p* = .227 for *work-life balance* respectively*.* Additionally, there was minimal difference in mean work pressure levels across the three categories of bedside nurses, *F* (2, 610) = 1.02, *p* = .363.

### 3.3 Path modeling

A path analysis was conducted to investigate the hypothesized relationships among the personal resources (work experience, need for autonomy, self-efficacy, and energy), home and job demands (parenthood, working hours, work pressure), contextual resource (work-life balance), and the intention to stay. The full model did not fit well (*χ*^2^(13) = 302.36; *p* < .001, CFI = .692, RMSEA = .190, SRMR = .072). Therefore, we followed the recommended model trimming by dropping non-significant relationships and adding correlations based on modification indices (Kline, 2023), between the need for autonomy and work-life balance, as well as between energy and work pressure. The final model optimally described the relationships among the factors. We then tested the indirect effects and 95% confidence intervals (CIs). Despite the model's complexity due to the number of observed factors, it fitted the data very well (*χ*2(16) = 38.39; *p* = .005, CFI = .976; TLI = .961, RMSEA = .048 (90% CI = .028; .067), SRMR = .030).

**Fig. 3:**
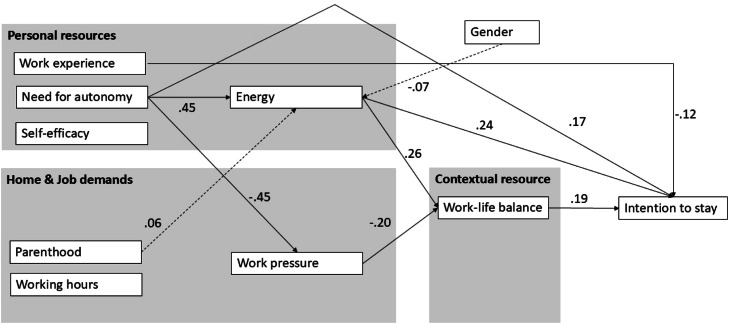
Path model of nurses' personal resources, home and job demands, work-life balance, and intention to stay Note. p < .05 dotted lines; p ≤ .001 otherwise.

**Fig. 4:**
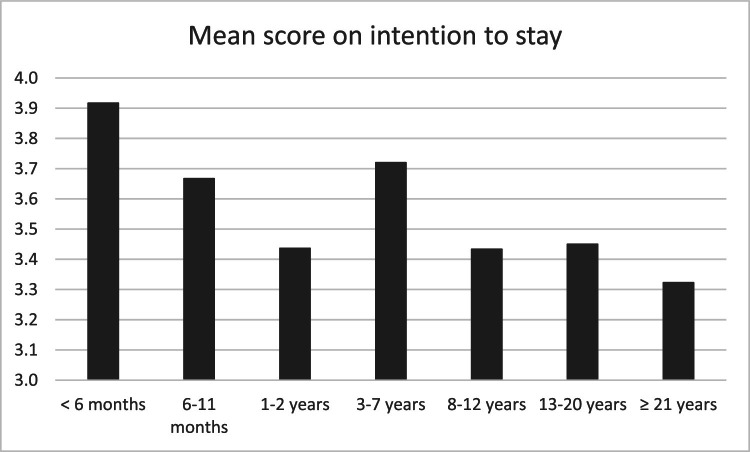
The mean score on intention to stay depending on years of experience.

Fig. 3 shows the final model with the significant relationships among standardized variables. We found significant positive relationships between need for autonomy and energy. The need for autonomy was negatively related to work pressure. Parenthood was positively related to energy, although the standardized effect was small. Energy was positively related to work-life balance, whereas work pressure was negatively related. The need for autonomy fulfilment, work-life balance, and energy were positively related to the intention to stay. Work experience was negatively related to the intention to stay. Fig. 4 shows that the relation between years of experience and the intention to stay was non-linear. Nurses with less than 11 months of experience and 3-7 years of experience had higher mean scores on the intention to stay compared to 1-2 years of experience or more than 8 years of experience. Work pressure was not directly related to the intention to stay. The model showed two significant indirect relationships via work-life balance between work pressure and the intention to stay (b*_indirect_ = -.04, CI [−.07; −.02]) and between energy and the intention to stay (b*_indirect_ = .05, 95% CI [.02;.08]). Furthermore, an indirect relationship was found between the need for autonomy and work-life balance via energy (b*_indirect_ = .12, 95% CI [.07; .17]). Self-efficacy and working hours did not appear to be beneficial personal resources in this context, as they were not significantly related to energy, work pressure, work-life balance, or the intention to stay. This model explained 21% of the variance in energy, 27% of the variance in work pressure, 27% of the variance in work-life balance, and 24% of the variance in intention to stay.

## 4 Discussion

Our purpose was to gain a better understanding of 1) to what extent and how home and job demands and personal and contextual resources related to work-life balance, and 2) how these related to the intention to stay in the nursing profession. We thereby based our classification of demands and resources on the Work-Home Resources model (Ten Brummelhuis & Bakker, 2012). We found that intention to stay among nursing staff was related to a combination of home demands (parenthood), job demands (work pressure), and personal and contextual resources (work experience, need for autonomy, and work-life balance). Energy was positively related to work-life balance, while work pressure was negatively related to it. In turn, work-life balance played a crucial role in nurses' intention to stay in the profession, together with the need for autonomy fulfilment. From the results, we have underscored the importance of addressing work and personal resources to improve both work-life balance and retention in nursing. The four key finding of this study are discussed in light of the literature below.

Firstly, consistent with previous research, we highlighted that energy positively related to nurses’ work-life balance, while work pressure was negatively related. Work demands, such as workload, shift work, and overtime, were strongly associated with work-life interference among nurses (Raffenaud et al., 2019). A lack of work-life balance, often resulting from work-family conflict and high workloads, may lead nurses to consider leaving the profession to protect their energy levels and health (Yildiz et al., 2021). Fair workload perceptions and the ability to balance work and family responsibilities may be crucial for achieving sustainable work-life balance, which reduces job stress and enhances retention (Labrague et al., 2021). Ultimately, promoting work-life balance through supportive policies and addressing excessive work demands is deemed essential for retaining nursing staff and fostering a sustainable workforce (De Vries et al., 2023; Matsuo et al., 2021; Riedl & Thomas, 2019).

Secondly, we have emphasized the relationship between fulfilling the need for autonomy and the intention to stay and in shaping nurses’ work-life balance indirectly via energy. More specifically, we found that the need for autonomy was positively associated with energy levels and retention. This supported researchers who linked autonomy to job satisfaction, better patient outcomes, and lower turnover intentions (Cziraki et al., 2020; De Vries et al., 2023; Wong, 2024). We have also aligned our findings with researchers who showed that nurses who leave the profession often experienced less autonomy compared to those who stayed (Kerzman et al., 2020) and that autonomy reduced work-family conflict (Sirgy & Lee, 2018). Beyond finding support for these patterns, we added to the literature by emphasizing autonomy's role as an antecedent in the relationship between work demands, energy, and retention. For instance, Cheng et al. (2019) showed that work-life interference mediated the relationship between perceived work quality and turnover intention. We contributed by showing that fulfilling the need for autonomy can buffer work demands to sustain energy and work-life balance. Together, we have underlined the possible importance of organizational policies that prioritize autonomy to support nurses’ well-being, professional commitment, and intention to stay.

Thirdly, contrary to expectations, we showed that parenting was associated with slightly increased energy levels. Parenting demands may foster a sense of purpose, responsibility, and structure, enhancing psychological capital—hope, self-efficacy, resilience, and optimism (Hazan-Liran, 2024; Nolzen, 2018)—which might help parents manage stress and sustain higher energy. Additionally, Dutch healthcare organizations' flexible work arrangements and the prevalence of part-time work (CBS, 2022) support parents in achieving work-life balance and maintaining energy levels. Parenthood might also provide energy due to increased life satisfaction, with less impact on work. Role accumulation theory posits that balancing multiple roles, such as employee and parent, promotes personal growth and social support, further boosting energy (Ma et al., 2022; Carlson & Kacmar, 2000). In contrast, non-parents may disproportionally encounter hidden energy drains, like informal caregiving or increased work-role overload, which can impact their mental health and lower their energy levels. Future researchers should account for informal caregiving responsibilities undertaken by nurses of all ages, parents as well as non-parents, as providing such care can significantly impact their energy levels and overall work performance (Ervin et al., 2022).

Fourthly, we revealed a nuanced relationship among work experience, energy, work-life balance, and the intention to stay in the nursing profession. We showed that increased work experience was associated with a lower intention to stay. However, further analysis revealed a more nuanced picture: nurses with less than 1 year of experience and those with 3-7 years scored higher, on average, on their intention to stay. This contrasted with researchers showing higher retention rates among senior nurses (Pressley & Garside, 2022; Van der Heijden et al., 2018). Less experienced nurses, despite facing risks of burnout and distress (Jarden et al., 2021; La Torre et al., 2021), may exhibit strong retention intentions due to early career enthusiasm, growth opportunities, or vitality (Lee, 2021; Moloney, 2024). Generational differences may impact work-life balance, job satisfaction, and retention decisions across experience levels (Tan & Chin, 2023; Wong, 2024). From these findings, we have underscored the possible need for tailored strategies to support nurses across different stages of their careers (Al Yahyaei et al., 2022; Van der Heijden et al., 2020). Future researchers should further explore these dynamics to better inform retention strategies.

We did not find an effect for the type of contract regarding working hours and the indirect link with work-life balance. Most nurses worked part-time and may have reduced their working hours to manage work and home demands. Previous researchers have indicated that women in the Netherlands use part-time work as a strategy to balance work and care responsibilities (Yerkes & Hewitt, 2019). Female nurses, who traditionally take on more home care responsibilities, may leave the profession due to home care demands (Tolksdorf et al., 2022). Future researchers can use a qualitative design to gain a deeper understanding of the patterns in home care and work-related responsibilities, the type of contract, and the intention to stay.

Our findings aligned with the Work-Home Resources model (Ten Brummelhuis & Bakker, 2012), indicating that personal resources, such as autonomy, may contribute positively to energy levels and work-life balance. Traditionally, the Job Characteristics Model of work motivation (Hackman & Oldham, 1974) has focused on outcomes like low absenteeism and turnover. In contrast, the Work-Home Resources and Job Demands-Resources model emphasize both negative outcomes, like health issues, and positive outcomes, such as team and individual performance. Although the Work-Home Resources model includes long-term outcomes, it did not initially center on the intention to stay (Ten Brummelhuis & Bakker, 2012).

Overall, we have contributed to the literature by demonstrating the Work-Home Resources model's utility in explaining long-term outcomes, such as retention in the nursing profession. Moreover, unlike prior researchers who focused on novice nurses, we included a full age range, emphasizing the importance of retaining both experienced and novice nurses. Finally, using path modeling, we explored the interrelationships among personal resources, home and job demands, work-life balance, and retention intention within a Western European context, where researchers focusing on retention remain scarce (e.g., Van der Heijden et al., 2019).

## 5 Limitations

We considered several limitations in this study. Firstly, its cross-sectional design precluded causal inferences. We recommend replicating the study in a longitudinal design to provide insights into changes over time in energy, work pressure, work-life balance, and the intention to stay. This approach would also distinguish between daily and long-term outcomes, as depicted in the Work-Home Resources model (Ten Brummelhuis & Bakker, 2012). Secondly, while the quantitative analysis clarified to what extent energy, work pressure, and work-life balance related to the intention to stay, it did not explain how experiences and circumstances influenced these variables. A qualitative design, such as an interview or diary study, is recommended (Merriam & Tisdell, 2016; Kunnen et al., 2024). Thirdly, given the large sample size and representative gender distribution, the results seem to be generalizable to the broader population of nurses in the Netherlands. However, there may be response bias, as the most motivated nurses are more likely to have participated, potentially skewing the results toward those with a higher intention to stay (cf. Menachemi, 2010). Future researchers should replicate the study with different samples across various institutions and countries to validate the findings. Fourthly, participants used an anonymized online survey link, which makes it impossible to fully eliminate the risk of repeated participation. To minimize this, we checked IP addresses, location data, and response patterns in relation to background characteristics. Lastly, we derived some of the item scales from other questionnaires tested in the workplace instead of healthcare institutions. Although the resulting questionnaire had face validity, we did not test it for content, construct, or other types of validity. This may affect the findings. However, we tested the internal consistency of the scales and provided the Cronbach's alpha.

## 6 Conclusions

Nurses’ fulfillment of the need for autonomy was associated with increased energy levels and, hence, work-life balance and the intention to stay in the profession. A similar, yet smaller, positive effect was found for parenthood. While the need for autonomy seems to reduce work pressure, work pressure was negatively associated with work-life balance. Additionally, more experienced nurses were found to be less likely to stay in the profession, suggesting that targeted support to address potential burnout and unmet expectations may be needed for long-serving nurses. Notably, the type of contract (e.g., part-time or full-time work) and self-efficacy were not significantly associated with energy or work pressure. Enhancing work-life balance may be crucial for improving retention rates among nurses. A supportive work environment may help reduce home and job demands, allowing nurses to maintain control and improve work-life balance (Navajas-Romero et al., 2020). Organizations could consider prioritizing providing nurses with greater autonomy and control over their tasks and schedules, as meeting the need for autonomy may enhance energy levels, reduce workload, and improve work-life balance, thereby fostering the intention to stay.

## Statement Data Sharing

Pseudonomized data is available at request. Participants did not explicitly provide informed consent to store their data in a database. It can be used only for other related research questions based on personal requests and in collaboration with the first author. Surveys and output files can be shared.

## CRediT authorship contribution statement

**Jasperina Brouwer:** Writing – review & editing, Writing – original draft, Project administration, Methodology, Investigation, Funding acquisition, Formal analysis, Data curation, Conceptualization. **Stéfanie André:** Writing – review & editing, Writing – original draft, Investigation, Conceptualization. **Nienke Renting:** Writing – review & editing, Investigation, Conceptualization.

## Declaration of competing interest

The authors declare that they have no known competing financial interests or personal relationships that could have appeared to influence the work reported in this paper.

## Appendix Supplementary materials

Image, application 1
