# Supplementary material for: The interplay between home and job demands, resources, and the intention to stay in nursing: A cross-sectional study
Source: Int J Nurs Stud Adv. 2025 Mar 17;8:100318. doi: 10.1016/j.ijnsa.2025.100318 (PMC12002953; doi:10.1016/j.ijnsa.2025.100318)
Supplement: Supplementary file 1 [file mmc1.docx]

**Supplementary Material**

**Table S1**

*Survey Constructs and Measurement Scales*

| Construct | Survey item or scale | Answer options |
| --- | --- | --- |
| Gender | Which gender do you most identify with? | - Male - Female - Non-binary |
| Age | Age | - 18 -20 - 21-25 - 26-30 - 31-40 - 41-45 - 46-50 - 51-55 - 56-60 - 61-67 - Other, namely |
| Working hours | What type of employment contract do you hold? | - Full-time (≥ 0.8 FTE; ≥ 32h) - Part-time (< 0.8 FTE; < 32h) - Flexible - Other |
| Position | What is your current job title/position? | - Registered Nurse (RN) - Registered Nurse Manager (in a team) - Licensed Practical Nurse (LPN) - Secretary - Certified Nursing Assistant (CNA) - Certified Nutrition assistant - Head of the department - Other, namely |
| Work experience | How many years have you worked in the healthcare sector? | - Less than 6 months - 6-11 months - 1-2 years - 3-7 years - 8-12 years - 13-20 years - 21 years or longer |
| Sector | Which healthcare sector do you work in? | - Youth care - Hospitals and clinics - Elderly care - Home care - Mental health care - Disability care - Emergency care - Legal care - General practitioner care - Health services and event care - Maternity care - Other, namely |
| Parenthood | What is your current household situation? | - Single - Married or cohabiting without children living at home - Married or cohabiting and one or more children living at home - Single without children living at home - Single or more children living at home - Living with home mates (e.g., student house) - Other, namely |
| Need for autonomy | The following statements relate to your perception of autonomy at work. Please, indicate the extent to which you agree with each statement.   1. I feel like I can be myself at my job. 2. At work, I often feel like I have to follow other people’s commands. 3. If I could choose, I would do things at work differently. 4. The tasks I have to do at work are in line with what I really want to do. 5. I feel free to do my job the way I think it could best be done. 6. In my job, I feel forced to do things I do not want to do. | 1. Fully disagree 2. Disagree 3. Somewhat agree, somewhat disagree 4. Agree 5. Fully agree |
| Self-efficacy | The following questions concern your commitment to your work self-efficacy (belief in your own abilities). Choose the option that best reflects your initial response.   1. I believe I perform well in my job. 2. I believe that reading professional literature and undergoing training will enhance my work. 3. I feel confident in handling complex situations. 4. I am certain that experience and practicing improve my nursing skills. 5. I am confident that if my performance would be evaluated, I would receive a good score. 6. I have the necessary skills to perform my tasks well. 7. I am confident that I have sufficient knowledge to perform my job well. 8. I am capable of carrying out my tasks effectively. 9. I have mastered the skills required for my work. | 1. Fully disagree 2. Disagree 3. Somewhat agree, somewhat disagree 4. Agree 5. Fully agree |
| Energy | 1. I find it difficult to relax at the end of a working day. 2. At the end of a working day, I feel really fatigued. 3. After a working day, I frequently feel too fatigued to engage in any other activity. | 1. Fully disagree 2. Disagree 3. Somewhat agree, somewhat disagree 4. Agree 5. Fully agree |
| Work pressure | The following questions concern how often you experience work pressure.  Choose the option that best reflects your initial response.   1. Do you work under time pressure? 2. Do you have to deal with a backlog at work? 3. Do you have too little work? 4. Do you have problems with the workload? 5. Do you wish you could work at an easier pace? | 1. Never 2. Sometimes 3. About half of the time 4. Most of the time 5. Always |
| Work-life balance | 1. My work and personal demands are well balanced. 2. I can divide my time and attention well between home and work. | 1. Fully disagree 2. Disagree 3. Somewhat agree, somewhat disagree 4. Agree 5. Fully agree |
| Turnover intention | Do you ever think about taking another job? Indicate to what extent you agree with the following statements. Choose the option that best reflects your initial response.   1. I sometimes think about leaving the nursing profession. 2. I intend to change professions next year. 3. I intend to continue working as a nurse for at least the next three years”. | 1. Fully disagree 2. Disagree 3. Somewhat agree, somewhat disagree 4. Agree 5. Fully agree |
